# Hypothermically Stored Amnion Is Robust and Provides a Scaffold for Supporting Wound Healing by Retaining the Characteristics of Native Tissue

**DOI:** 10.3390/ijms251910347

**Published:** 2024-09-26

**Authors:** Katrina A. Harmon, Kelly A. Kimmerling, Justin T. Avery, Katie C. Mowry

**Affiliations:** Organogenesis Discovery Center, Birmingham, AL 35243, USA

**Keywords:** hypothermic storage, amniotic membranes, placental membranes, scaffold

## Abstract

Placental-derived products have been used since the early 1900s for wound applications and have shown clinical utility in supporting wound healing. A hypothermically stored amniotic membrane (HSAM) was developed using a proprietary process to allow for the retention of the extracellular matrix (ECM), viable cells, and key proteins. To evaluate its utility, we characterized the HSAM and compared it to a native unprocessed amniotic membrane (uAM) and a dehydrated amniotic membrane (dAM), as well as assessing the functionality of the HSAM as a scaffold to promote cell growth. The HSAM, uAM, and dAM were compared using scanning electron microscopy (SEM), histology, and thickness. Scaffold durability was assessed in vitro using mechanical testing and a simulated wound fluid (SWF) model. The ability of the HSAM to act as a scaffold was evaluated using an in vitro attachment model. The HSAM showed similar structural characteristics compared to the uAM; however, the dAM was significantly more compact. There were no significant differences between the HSAM and the uAM following degradation in an SWF model. ECM- and placental-related proteins were shared between the HSAM and uAM, and the HSAM enhanced the attachment and proliferation of fibroblasts in vitro. The HSAM is substantially similar to the uAM by retaining key regulatory proteins, resisting degradation in SWF, and acting as a scaffold for cellular growth and invasion.

## 1. Introduction

Wound healing, or the process by which skin integrity is restored, consists of four overlapping phases—hemostasis, inflammation, proliferation, and remodeling [1]. Hemostasis begins immediately following injury, resulting in the constriction of blood vessels, which, in turn, activates platelets [2]. This activation results in the secretion of pro-inflammatory cytokines, such as interleukin-1 and tumor necrosis factor-alpha, along with transforming growth factor-beta (TGF-β), initiating the inflammatory phase [1,2]. These factors attract immune cells, such as monocytes and neutrophils, which are essential for advancing to the proliferative phase through the recruitment of fibroblasts and keratinocytes [1,2]. Fibroblasts produce a provisional matrix, leading to wound closure and epithelialization [1,2], which is remodeled over time.

In chronic wounds, the wound healing process often stalls in the inflammatory phase, due, in part, to the increased levels of matrix metalloproteinases (MMPs) and pro-inflammatory cytokines [3,4]. The imbalance between MMPs and their inhibitors leads to the destruction of newly formed extracellular matrix (ECM), preventing the advancement of the wound through the healing cascade [4]. Additionally, the aberrant inflammation found in chronic wounds can lead to bacterial infection and biofilm formation [5,6]. Due to the high cost of treating wounds, in which Medicare cost projections in 2018 ranged between USD 28.1 and USD 96.8 billion for all wound types [7], there is a need for a treatment that provides a scaffold for tissue growth and promotes the transition from the inflammatory phase to the proliferative and remodeling phases.

Placental tissues have been used since the early 1900s for a variety of applications, including burns and wounds [8,9,10]. Recently, clinical studies have shown the utility of fresh amniotic membrane for the treatment of a variety of wounds [11,12,13,14,15], and preclinical work has shown that amniotic membranes contain growth factors and cytokines, providing a scaffold for fibroblast attachment, migration, and growth [16,17,18]. Fresh amniotic membranes allow for the retention of water in the ECM, preserving a physical scaffold architecture that has been reported by Badylak to be beneficial for scaffolds in supporting cell migration, attachment, and proliferation [19]. The retention of a native ECM structure may also maintain the innate remodeling capabilities of native ECM scaffolds [19]. Furthermore, the preservation of the natural state of scaffolds is expected to allow for increased durability and the retention of key regulatory proteins that aid in supporting matrix remodeling and cellular growth [19,20].

A hypothermically stored amniotic membrane (HSAM) was developed, consisting of amniotic membrane that is processed using a proprietary process to allow for the preservation of the native ECM structure, along with the retention of viable cells and regulatory proteins. We hypothesized that the proprietary process utilized preserves the HSAM, maintaining the key characteristics and functionality of the native unprocessed amniotic membrane (uAM). In this work, we compared the HSAM to a donor-matched uAM and dehydrated amniotic membrane (dAM) to determine whether the HSAM retains the mechanical integrity, durability, and regulatory proteins found in the uAM. We further assessed the utility of the HSAM to function as a scaffold for the cellular attachment and proliferation of human dermal fibroblasts, an important cell type for wound healing.

## 2. Results

### 2.1. Hypothermic Storage Maintains Native Amnion Tissue Structure and Integrity

**Figure 1:**
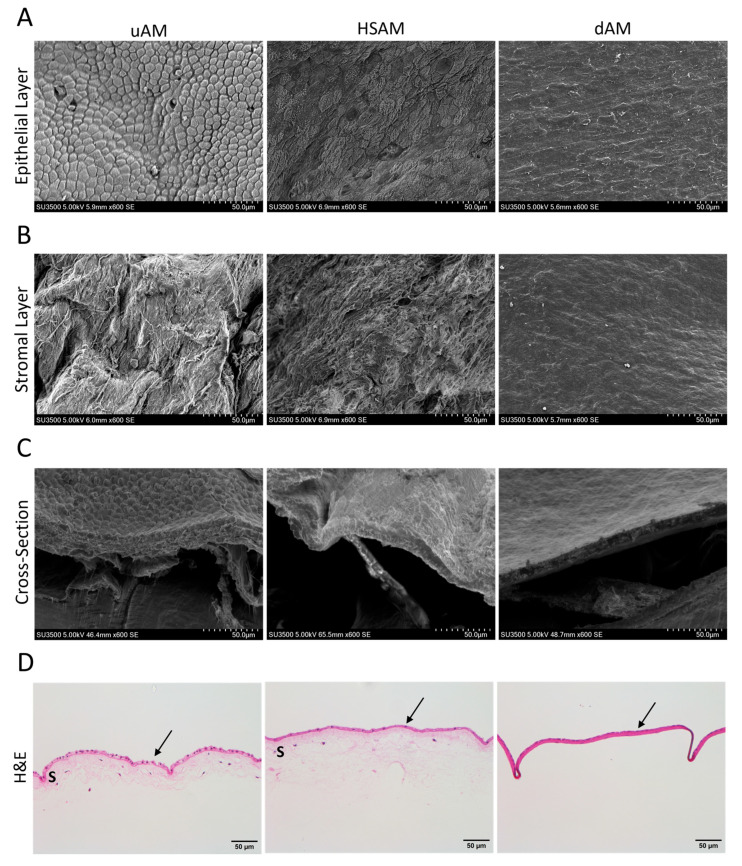
Characterization of tissues by scanning electron microscopy (SEM) and histology. Representative SEM images of the (**A**) epithelial and (**B**) stromal layers, along with (**C**) a cross-sectional view. (**D**) Representative hematoxylin and eosin (H&E) staining of tissues. Magnification for SEM = 600×; H&E = 20×; scale bars for images are 50 µm. uAM: unprocessed amniotic membrane; HSAM: hypothermically stored amniotic membrane; dAM: dehydrated amniotic membrane; arrow: epithelial layer; S: spongy layer.

The characterization of processing techniques between the uAM, HSAM, and dAM was first assessed using SEM techniques (Figure 1A–C). The uAM showed a well-established and intact layer of epithelial cells (Figure 1A), with ECM fibers evident in the stromal layer (Figure 1B). The HSAM images mirrored the uAM on both the epithelial and stromal layers, while the dAM resulted in a less observable epithelial layer and indistinguishable ECM fibers due to compaction as a result of water loss during dehydration (Figure 1A,B). The evaluation of the cross-sectional SEM images showed that both the uAM and HSAM had similar thicknesses and structure, while the dAM was compressed in comparison (Figure 1C). H&E-stained images corresponded with SEM images, showing a lightly colored, larger area of ECM in the uAM and HSAM samples compared to a thick, dark layer of dense ECM in the dAM (Figure 1D).

**Figure 2:**
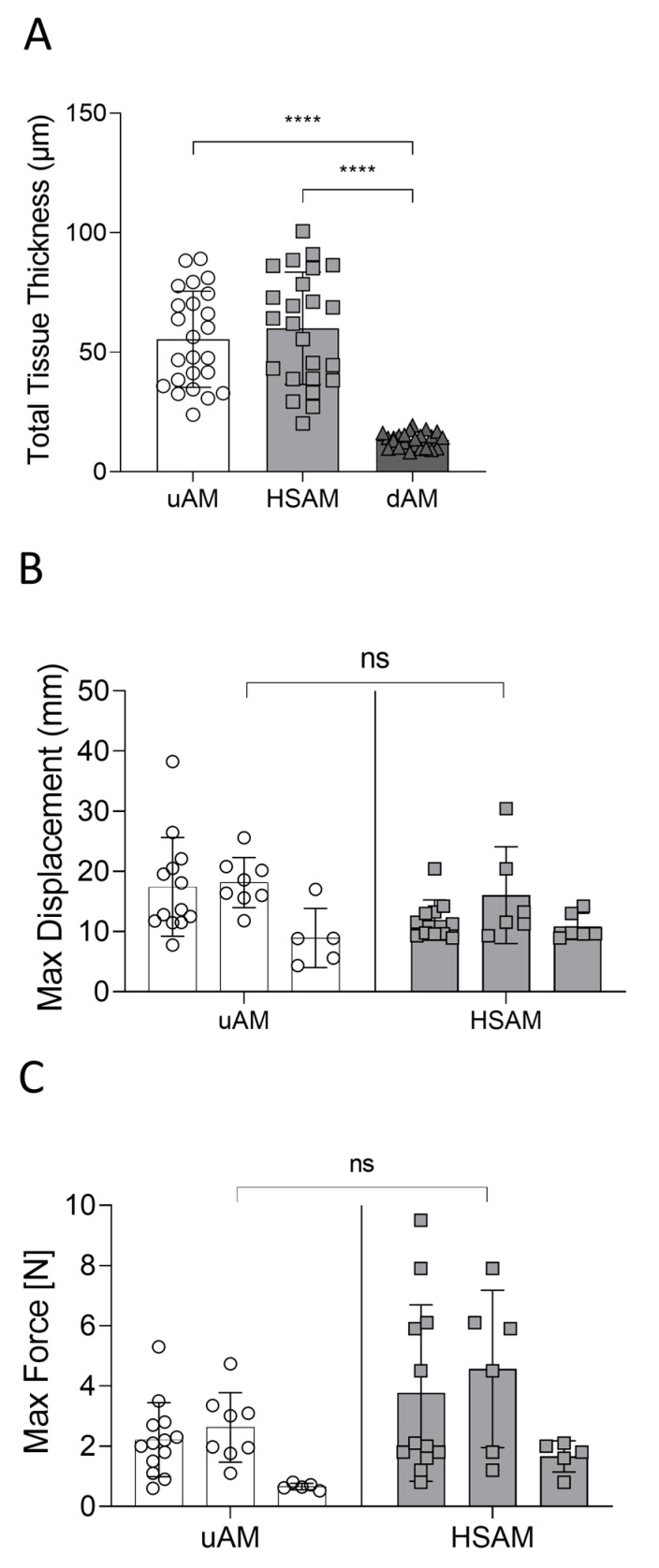
Characterization of tissues by thickness measurements and tensile testing. (**A**) Total thickness measurements in the uAM, HSAM, and dAM following processing. (**B**) Maximum displacement and (**C**) maximum force for the uAM and HSAM. Average ± standard deviation reported; **** denotes *p* < 0.0001. uAM: unprocessed amniotic membrane; HSAM: hypothermically stored amniotic membrane; dAM: dehydrated amniotic membrane; ns: not significant.

H&E images were used to quantify the thickness between processing techniques (Figure 2A). The uAM and HSAM resulted in similar thicknesses (*p* = 0.66), while the dAM resulted in a significant decrease in graft thickness compared to both the uAM and HSAM (*p* < 0.0001 for both). Additionally, tensile testing was conducted to assess the tissue integrity between the uAM and HSAM (Figure 2B,C). Of note, the dAM was not assessed due to initial pilots confirming the brittle nature imparted by dehydration. Because of this finding and the compressed nature of the dAM, it was subsequently omitted from all following studies. There were no differences between the uAM and HSAM for either displacement or maximum force (*p* = 0.4881 and *p* = 0.1906, respectively). Though not significant, inter- and intra-variability between donors was observed, as expected.

### 2.2. Hypothermically Stored Amnion Maintains Durability over 17 Days

**Figure 3:**
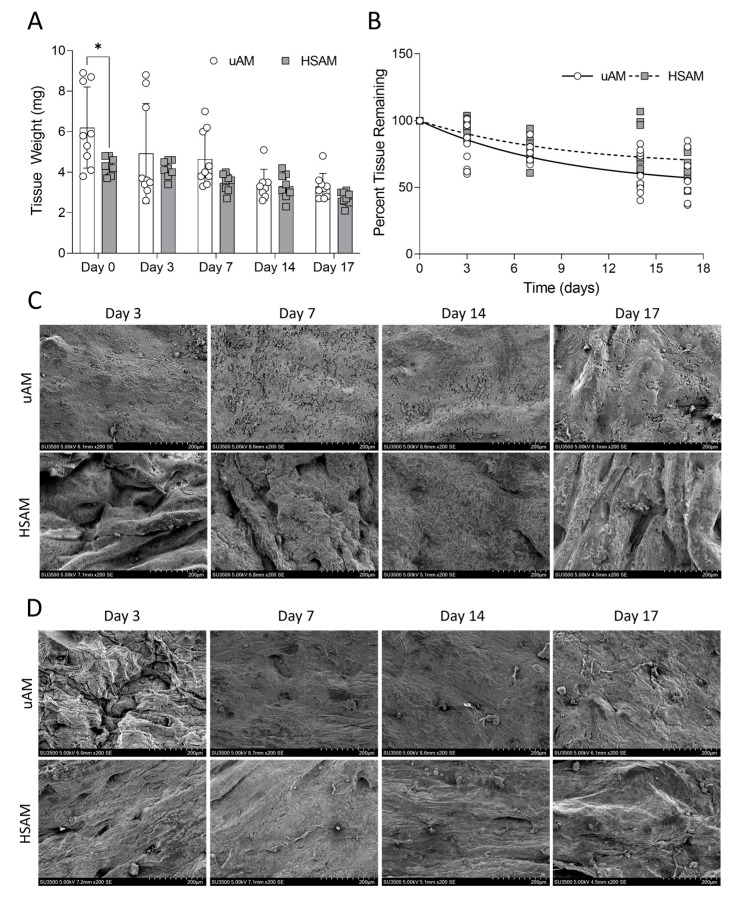
Durability of tissues in simulated wound fluid (SWF). (**A**) Tissue weight of remaining tissue and (**B**) rate of degradation after 17 days in SWF. Representative scanning electron microscopy (SEM) images of changes in the (**C**) epithelial and (**D**) stromal layers following degradation in SWF. Average ± standard deviation reported; * denotes *p* < 0.05. Magnification for SEM = 200×; scale bars for images are 200 µm. uAM: unprocessed amniotic membrane; HSAM: hypothermically stored amniotic membrane.

Differences in durability between the uAM and HSAM were evaluated using an in vitro SWF model to mimic a wound environment for up to 17 days (Figure 3A). At day 0, the uAM had a significantly higher tissue weight compared to the HSAM (*p* = 0.0181); however, at all other time points, there were no significant differences between the uAM and HSAM. Overall, both the uAM and HSAM retained over 50% of their initial tissue weight over the 17-day study, with no significant difference in the rate of degradation (*p* = 0.0900; Figure 3B). Representative SEM images of the epithelial side (Figure 3C) and stromal side (Figure 3D) are shown following degradation in SWF. On the epithelial side, a gradual loss of cellular continuity was observed in both the uAM and HSAM at as early as day 3 of exposure in SWF, resulting in gaps compared to the non-degraded samples (Figure 1A). ECM fibers were also slowly lost over time in the spongy layer compared to non-degraded uAM and HSAM samples (Figure 1B) following incubation with SWF.

### 2.3. Hypothermically Stored Amnion Maintains Scaffold Characteristics and Function

**Figure 4:**
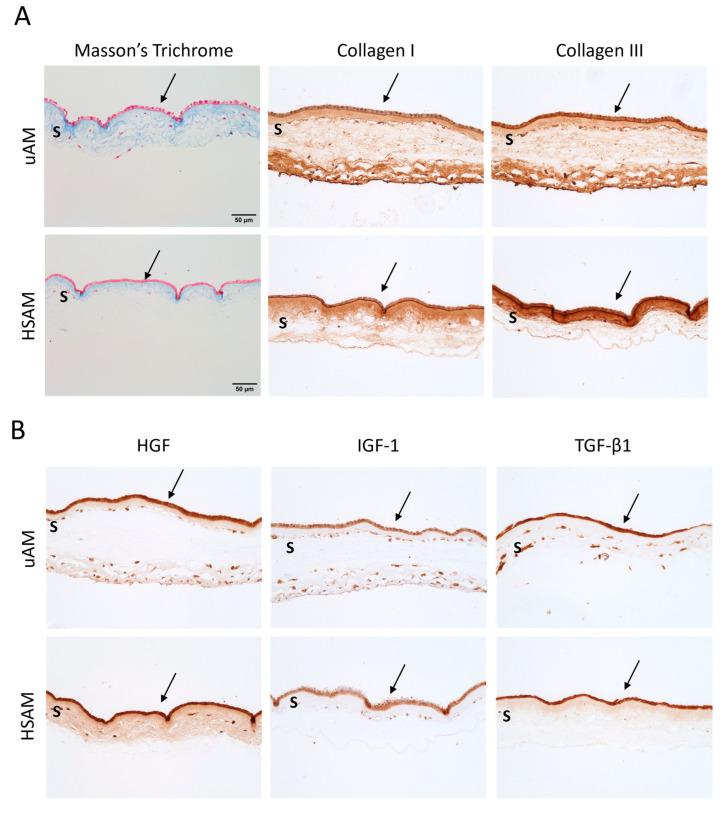
Characterization ECM properties and functionality as a scaffold. Representative (**A**) Masson’s Trichrome staining and immunohistochemistry (IHC) of relevant extracellular matrix markers and (**B**) IHC of relevant placental-related growth factors. Magnification for Masson’s Trichrome, IHC, and H&E: 20×; scale bars for all images are 50 µm. uAM: unprocessed amniotic membrane; HSAM: hypothermically stored amniotic membrane; arrow: epithelial layer; S: spongy layer; HGF: hepatocyte growth factor; IGF-1: insulin-like growth factor 1; TGF-β1: transforming growth factor beta 1.

An assessment of matrix proteins and growth factors and cytokines was carried out using two distinct staining techniques. Broadly, Masson’s Trichrome staining revealed similar red keratin staining and blue collagen staining in both the uAM and HSAM samples (Figure 4A). Next, IHC staining for specific ECM proteins showed collagen I and III distributed throughout both uAM and HSAM tissues (Figure 4A). Collagen I was present throughout the spongy layer, while collagen III was primarily localized to the basement membrane and spongy layer. Growth factors and cytokines, including hepatocyte growth factor (HGF), insulin-like growth factor 1 (IGF-1), and TGF-β1, were present in both the uAM and HSAM, and were highly concentrated in the basement membrane (Figure 4B).

**Figure 5:**
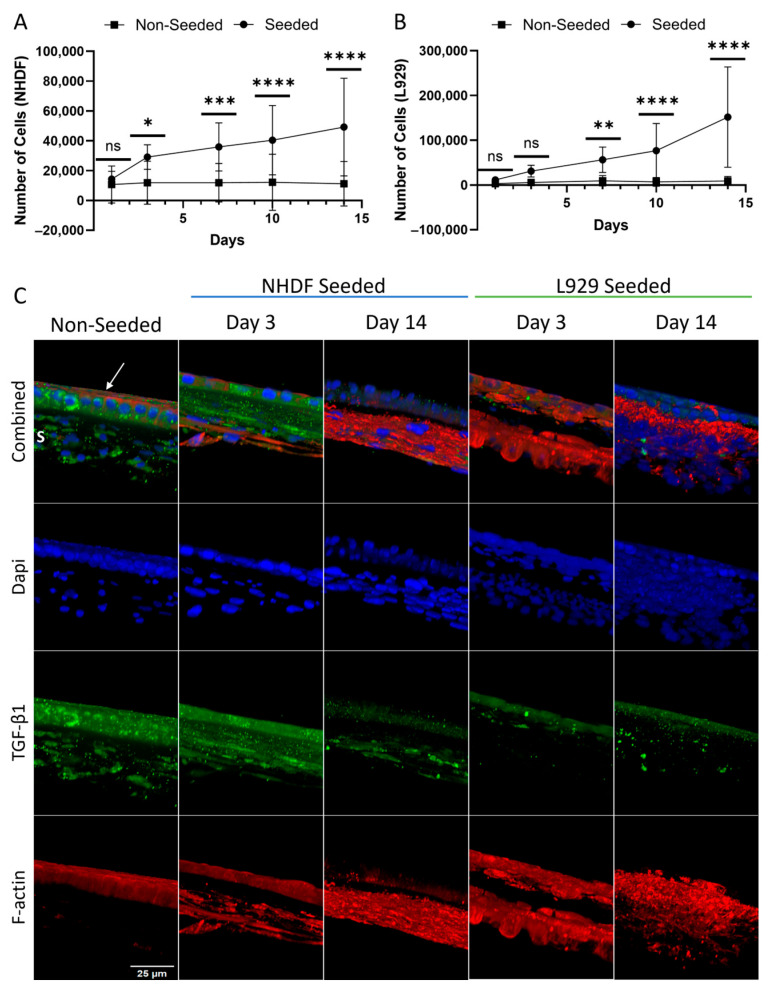
Fibroblast attachment and proliferation following seeding with human dermal fibroblasts (NHDF) and mouse fibroblasts (L929) onto hypothermically stored amniotic membranes. Cell number from AlamarBlue assessment showing metabolic activity of NHDFs (**A**) and L929s (**B**). (**C**) Representative immunofluorescence staining of seeded and non-seeded scaffolds at day 3 and 14; 40× magnification. Average ± standard deviation reported; ns denotes not significant; * denotes *p* < 0.05; ** denotes *p* < 0.01; *** denotes *p* < 0.001; **** denotes *p* < 0.0001 compared to non-seeded controls. Blue: nuclei; green: TGF-β1; red: f-actin (phalloidin); arrow: epithelial layer; S: spongy layer; Scale bar: 25 µm.

The utility of the HSAM as a scaffold was evaluated using an in vitro attachment model utilizing two fibroblast cell lines, a human dermal fibroblast cell line, and a mouse fibroblast cell line (L929s); of note, the uAM was not assessed in this study for scaffold functionality due to its known biological function as a scaffold during development. Seeding with human dermal fibroblasts showed a significant increase in cell number compared to non-seeded controls at days 3, 7, 10, and 14 (*p* < 0.05 for all; Figure 5A), while mouse fibroblasts showed a significant increase in cell number compared to non-seeded controls at days 7, 10, and 14 (*p* < 0.05 for all; Figure 5B). Representative immunofluorescent imaging (Figure 5C) visually depicts human and mouse fibroblast attachment and proliferation over time on the stromal-seeded side, as demonstrated by increased cell nuclei and f-actin staining from day 3 to day 14 compared to non-seeded controls. Furthermore, TGF-β1 was primarily concentrated in the epithelial layer and basement membrane of non-seeded and fibroblast-seeded grafts with expression reduced over time.

**Figure 6:**
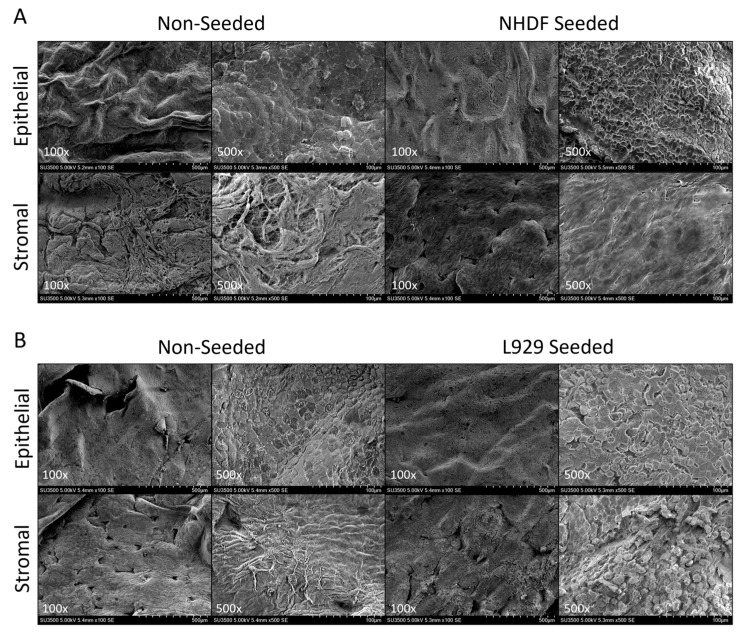
Structural characterization of fibroblasts seeded onto hypothermically stored amniotic membranes. Representative scanning electron microscopy images of non-seeded and human fibroblasts seeded (**A**) and mouse fibroblast seeded (**B**) grafts. For SEM, representative images are at 100× (scale bar: 500 µm) and 500× (scale bar: 100 µm).

SEM imaging (Figure 6A) showed the deposition of collagen from human fibroblast cells as depicted by the reduction in the ability to visualize the basket-weave texture shown in the non-seeded controls, while the epithelial layer (non-seeded side) of seeded grafts appeared comparable to their non-seeded controls. Mouse fibroblasts seeded onto the HSAM (Figure 6B) show the presence of cells on the stromal side (seeded side) compared to the non-seeded controls, while the epithelial layer (non-seeded side) appeared similar between the seeded and non-seeded grafts.

## 3. Discussion

Compared to the uAM, the HSAM was found to exhibit similar epithelial and stromal layer characteristics, including cellularity and ECM fibers, using SEM imaging; this was also confirmed with H&E staining. In contrast, the dAM showed compact, dense layers with a loss of details due to water loss during dehydration. Quantitative thickness measurements showed similar values for the uAM and HSAM, which were both significantly thicker than the dAM. There were no significant differences in tensile strength between uAM and HSAM, and the evaluation of degradation in SWF over 17 days yielded similar results. Finally, the uAM and HSAM shared ECM- and placental-related proteins, as shown by Masson’s Trichrome and IHC. The assessment of HSAM as a scaffold to support cell growth resulted in a significant increase in cell proliferation from day 3 to day 14 with human fibroblasts and from day 7 to day 14 with mouse fibroblasts. Taken together, these data support the hypothesis that hypothermic storage results in the maintenance of key characteristics of fresh amniotic membrane and suggest that the HSAM may effectively support the natural progression of wounds through the healing cascade.

In this study, initial characterization included the use of a native uAM and dAM, as research has shown that processing techniques can affect overall tissue properties. For instance, a previous study comparing unprocessed versus dehydrated amnion membranes showed a loss in thickness and over 51% of the growth factor and cytokine content following dehydration [17]. Because of these changes in growth factor and cytokine concentration, the dAM was not further assessed in this study following initial characterization assays. In addition, comparison of the uAM to the HSAM was necessary to ensure that the processing did not result in the loss of the critical characteristics of the uAM, such as the ECM structure and composition of key regulatory proteins. Published studies have shown the presence of ECM-related proteins, such as collagen I and collagen III, along with placental-related proteins such as TGF-β1 in native amniotic membrane [18,21,22], which were all found within the HSAM grafts. One noted limitation of the use of fresh amniotic membrane is the biodegradation of tissue [22]; the proprietary processing method used for the HSAM allowed for the retention of these key characteristics for up to 42 days.

In addition to preserving the key characteristics of the uAM, the durability of the HSAM was assessed to understand how grafts would naturally degrade in a wound environment to minimize the need for frequent applications to support healing. The degradation of the uAM and HSAM were similar, with both resisting rapid degradation in vitro. This model used a simulated wound fluid; however, we expect degradation of both the uAM and HSAM to be accelerated in chronic wounds, which often contain high levels of MMPs and inflammation [1,4,23]. A significant difference in tissue weight was observed at day 0; however, these tissues were donor-matched, so this finding could be due to differences in thicknesses within the amniotic membrane [22]. However, inter-placental variability has been shown to not affect the healing efficacy of amniotic membranes in treating chronic wounds [24].

The architecture and biological factors that promote remodeling using ECM scaffolds are anticipated to be the same factors that have evolved in vivo in native tissues to support homeostasis and repair following injury [19]. The assessment of the HSAM for the maintenance of key ECM proteins and growth factors from the uAM confirmed the presence of collagens, along with placental-derived factors such as HGF and IGF-1, which have been shown to promote fibroblast and keratinocyte migration and proliferation [25,26]. A previous study in a full-thickness rat model showed that the HSAM promoted epidermal formation and a basket-weave matrix like unwounded skin, expected to be due, in part, to the open ECM, allowing for better cell migration and attachment to the graft [27]. Fibroblasts cultured on the HSAM grafts for 2 weeks have previously shown that they will attach, invade, and remodel the matrix [16], and these results were confirmed herein, along with quantification of the rate of proliferation.

Clinically, the HSAM grafts have been shown to be effective in supporting healing in chronic wounds. In a randomized controlled trial for the treatment of diabetic foot ulcers, treatment with the HSAM resulted in significantly greater wound closure at weeks 12 and 16 compared to the standard of care [12]. In a separate single-arm clinical study for venous leg ulcers (VLUs), the response of VLUs and the wound microenvironment to the HSAM grafts showed that the presence of the HSAM in the wound bed was almost absent by seven days following treatment, highlighting the rapid remodeling initiated by the HSAM grafts; a total of 53% of subjects had complete re-epithelialization by week 12 [13]. Interestingly, levels of MMP-7, MMP-10, and tissue inhibitor of metalloproteinase-4 were significantly increased in wounds on a healing trajectory [13]; research has shown that the presence of MMP-7 and MMP-10 are required for remodeling, as MMP-10 is secreted by the epithelial layer during cell migration [23]. Although MMPs are necessary as part of the wound healing process, aberrant or prolonged expression is known to be a major factor in non-healing wounds [4,13,23].

Taken together, the data presented in this manuscript, along with previous clinical work, suggest a role for the HSAM in supporting wound healing due to its ability to retain the ECM structure of native unprocessed amniotic membrane and key regulatory proteins, capacity for durability within a modeled wound environment, and functionality as a scaffold for cells to attach to, proliferate on, and invade into. Future work evaluating the durability of the HSAM in an SWF environment with the addition of proteases, along with assessing the functionality of the ECM scaffold as it degrades over time, will allow for an additional mechanistic understanding of how skin substitutes support healing and contribute to new innovations.

## 4. Materials and Methods

### 4.1. Human Amniotic Tissue Sourcing and Processing

Donated placentas from planned cesarean sections were utilized, and all tissue processes were completed according to the American Association of Tissue Banks and the Food and Drug Administration’s Good Tissue Practices. Because there is no intervention or interaction with individuals, and the donated tissues are de-identified, this research does not involve human subjects and does not require institutional review board approval.

The amniotic membrane was separated from the chorion membrane; then, amniotic tissue was processed as three separate groups—uAM, HSAM, and dAM. Following processing, the uAM was utilized within 24 h, the dAM was dried overnight prior to use, and the HSAM was aseptically processed using a proprietary processing method (AlloFresh™, Organogenesis, Canton, MA, USA) and stored at 1–10 °C for up to 42 days until use. Three independent tissue donations were acquired and used for all experiments herein, except where mentioned below.

### 4.2. Tissue Characterization

Characterization of the different processing methods was carried out using scanning electron microscopy (SEM). The uAM, dAM, and HSAM were fixed in 3% glutaraldehyde, dehydrated to 100% ethanol, and dried in a Denton DCP-1 critical point dryer (Denton Vacuum, Moorestown, NJ, USA). Once dried, aluminum stubs were used to mount samples, followed by gold palladium coating using a Hummer 6.6 Sputter Coater (Anatech USA, Sparks, NV, USA) in an argon saturated environment. After sample preparation, imaging was acquired using a Hitachi SU3500 SEM (Hitachi High-Tech America Inc., Schamburg, IL, USA) at 5 kV at a magnification of 600×.

The histological assessment of tissues was carried out by fixing tissues in 4% paraformaldehyde (Thermo Scientific Chemicals, Waltham, MA, USA), followed by processing using paraffin embedding. Then, 5 µm serial sections were cut and floated onto charged glass slides, and were then dried overnight. Standard histologic stains, including hematoxylin and eosin (H&E) and Masson’s Trichrome were used to assess matrix and cellular characteristics. All histological images were captured at a magnification of 20× on an inverted microscope (Nikon Eclipse Ti, Nikon, Melville, NY; EVOS M5000, Thermo Fisher, Waltham, MA, USA).

Following histological imaging, tissue thickness was assessed by stitching together H&E images taken at 10× magnification and dividing images into equal grids. From there, grids were numbered sequentially, and used via random selection to acquire measurements in ImageJ v1.54g (National Institutes of Health, Bethesda, MD, USA). Three independent measurements from each grid were taken, with all measurements averaged to give the total graft thickness per image.

### 4.3. Tensile Testing of Tissues

To assess tissue integrity, the uAM and HSAM were cut into 2 cm × 7 cm rectangles for testing, with gauze soaked in phosphate-buffered saline placed onto the ends of each piece of tissue. A 3 cm separation of the pneumatic grips allowed for 1 cm of tissue to be placed into each end of an Instron Model 3342 (Instron, Norwood, MA, USA). Force was applied to the samples using a 50 N load cell, and the displacement and maximum force for each tissue sample was obtained. At least *n* = 5 per donor was tested to account for inherent tissue variability.

### 4.4. Tissue Durability in an In Vitro Simulated Wound Fluid Assay

To assess the durability and degradation of the uAM and HSAM, an in vitro simulated wound fluid (SWF) model was developed. SWF was prepared as previously described [28], and 2 cm^2^ of the uAM and HSAM were incubated in SWF at 37 °C for up to 17 days with gentle agitation. Every 2–3 days, SWF was replaced with fresh SWF. At each assessment time point, tissue samples were dried and weighed, with the percentage of tissue remaining calculated using the initial dry weights. Representative SEM images of tissues at each assessment time point were taken using the methods described herein.

### 4.5. Immunohistochemical Analysis of Key Components

Immunohistochemistry (IHC) of the uAM and HSAM was completed to evaluate the location and retention of various placental- and matrix-related growth factors and cytokines. Sections were prepared as described above, and were then processed through a gradient of ethanol to water. Antigen retrieval was either carried out by placing slides into a 0.01 M Tris + 1 mM ethylenediaminetetraacetic acid (EDTA) buffer at pH 9.0 for 20 min at 70 °C, or into a Proteinase K (1:50 dilution) + Tris EDTA-CaCl_2_ buffer at pH 8.0 for 15 min at 37 °C. Following antigen retrieval, slides were washed with deionized water, transferred to a 0.05 M Tris + 0.15 M NaCl solution with 0.1% *v*/*v* Triton-X-100 at pH 7.6, then blocked using 3% hydrogen peroxide in endogenous peroxidase for 20 min. An additional block for 30 min using 3% normal goat serum was conducted prior to incubating slides with primary antibodies overnight at 4 °C. Following overnight incubation, slides were washed with Tris-buffered saline + Tween 20, and were then incubated with the relevant secondary antibody conjugated to horseradish peroxidase. The development of slides was carried out using diaminobenzidine and counterstained with hematoxylin. Then, 20× magnification images were taken using an inverted microscope (EVOS M5000, Thermo Fisher, Waltham, MA, USA).

### 4.6. Fibroblast Attachment and Proliferation Studies

#### 4.6.1. Cells, Media, and HSAM

Primary normal adult human dermal fibroblasts (NHDFs, lot 22TL073035 [female, age 46, Black], Lonza Biosciences, Walkersville, MD, USA) and two lots of primary mouse fibroblasts (L929, lots 14A015/70058860, ATCC, Bethesda, MD, USA) were thawed and cultured as per the manufacturer’s recommendations. For all experiments, growth media (GM) was used and consisted of 10% fetal bovine serum (FBS; Corning, Corning, NY, USA) and 1% antibiotic antimycotic solution (Corning, Corning, NY, USA) in Dulbecco’s Modified Eagle’s Medium (DMEM; Corning, Corning, NY, USA).

HSAM grafts (Affinity™, Organogenesis, Canton, MA, USA) were utilized. Grafts were individually secured with CellCrowns™ (Scaffdex, Tampere, Finland) with the stromal side facing up in a 12-well plate (Corning, Corning, NY, USA). A total of 50,000 cells per well in 50 μL of GM were seeded onto the stromal side and allowed to attach for a minimum of 3 h at 37 °C at 5% CO_2_. Following attachment, 2 mL of GM was added, and plates were incubated until assessment at days 1, 3, 7, 10, and 14. Media was changed every 2–3 days on all grafts.

#### 4.6.2. Quantitation of Proliferation

Grafts at assessed timepoints were fixed in 4% paraformaldehyde, permeabilized with 0.01% Triton X-100 for 20 min at room temperature, and blocked in 5% goat serum for 1 h at 37 °C. Grafts were incubated with TGF-β1 primary antibody (1:100; Thermo Fisher, Waltham, MA, USA) at 37 °C then incubated with Alexa Fluor 488-conjugated secondary antibody (1:500; Themo Fisher, Waltham, MA, USA) at 37 °C. DAPI (4′,6-diamidino-2-phenylinodole) was used to stain nuclei, while Rhodamine Phalloidin (1:100; Thermo Fisher, Waltham, MA, USA) was used to stain filamentous actin (f-actin). Representative images were captured at a magnification of 40× using a fluorescence confocal microscope (Nikon A1R, Nikon, Melville, NY, USA).

Proliferation was also visualized using SEM. HSAM grafts were fixed in 4% paraformaldehyde prior to immersion in 8% paraformaldehyde at 4 °C for two days. Images of non-seeded and fibroblast seeded HSAM at 14 days were taken at a magnification of 100× and 500× using the methods described above.

### 4.7. Statistical Analyses

Statistical analyses were performed with GraphPad Prism v10.3 (GraphPad Software, Boston, MA, USA). A one-way analysis of variance (ANOVA) with Tukey’s post hoc test was performed to determine statistical differences in tissue thickness. A nested *t*-test was used to evaluate differences in displacement and maximum force. A two-way ANOVA with Šidák’s post hoc test was performed to determine differences in in vitro SWF studies, while a one-phase decay fit was conducted and the Y0 was constrained to 100 in analyzing the rate of in vitro degradation. A two-way ANOVA with Šidák’s post hoc test was used to evaluate differences between time points for fibroblast attachment experiments. Outliers were removed using the ROUT method. Average ± standard deviation is reported for all graphs. Electronic laboratory notebooks were used.

## 5. Conclusions

In this study, the impact of processing techniques between an unprocessed, hypothermically stored, and dehydrated amniotic membrane were evaluated. Results demonstrated that the HSAM exhibited similar characteristics compared to the uAM, while dehydration resulted in significant tissue compression. Key findings include the HSAM maintenance of extracellular matrix composition and function including structure, proteins, strength, and cellularity. Additionally, HSAM resisted rapid degradation and supported fibroblast attachment and proliferation for up to 14 days. As a result, by maintaining the key properties of unprocessed, fresh amniotic membranes, these results highlight the role the HSAM can play in supporting wound healing.

## Data Availability

The data that support the findings of this study are available from the corresponding author upon reasonable request.
